# Challenges in measuring serum calprotectin in autoinflammation: a comparative study of different immunoassays

**DOI:** 10.1186/s40348-026-00267-8

**Published:** 2026-11-10

**Authors:** Helmut Wittkowski, Carolin Park, Melanie Saers, Sabrina Fühner, Susanne Schleifenbaum, Claas Hinze, Manfred Fobker, Andrea Karolin, Michael P. Horn, Bernhard Schlüter, Dirk Foell

**Affiliations:** 1 Department of Pediatric Rheumatology and Immunology, University Children’s Hospital Muenster, Albert-Schweitzer-Campus 1, W30, Muenster, 48161 Germany; 2 Central Laboratory, University Hospital Muenster, Muenster, Germany; 3 University Institute of Clinical Chemistry, University Hospital of Bern, Bern, Switzerland

**Keywords:** Autoinflammatory diseases, Calprotectin, S100A8/S100A9, Immunoassays, ELISA, PETIA, CLIA

## Abstract

**Background:**

Serum calprotectin (S100A8/A9 complex) is increasingly used in the clinical evaluation of autoinflammatory diseases (AID). However, systematic comparisons of commercially available assays remain limited. This study aimed to assess comparability, and clinical applicability of commercial calprotectin assays to support standardized biomarker-guided diagnostics.

**Methods:**

Serum samples from 40 individuals—systemic juvenile idiopathic arthritis (sJIA, *n* = 10), adult-onset Still’s disease (AOSD, *n* = 10), familial Mediterranean fever (FMF, *n* = 10), PAPA syndrome (*n* = 2), infections (*n* = 5), leukemia (*n* = 3), and healthy pediatric controls (*n* = 10)—were analyzed. Calprotectin concentrations were measured using four ELISAs (BÜHLMANN, R&D Systems, BioLegend, BMA), two PETIAs (sCAL turbo, GCAL), and one CLIA (QuantaFlash) and compared with a BÜHLMANN ELISA reference. ELISA assays were measured with a secondary 10-fold dilution, while PETIA and CLIA assays were performed on automated platforms with reflex dilution. Spiking experiments were conducted to evaluate recovery.

**Results:**

All assays except BMA (*r* = 0.52) showed strong correlation with the reference (*r* ≥ 0.96). Median values in healthy controls varied markedly (0.29–3.22 µg/mL), with inter-assay differences exceeding one log scale. Several assays underestimated very high concentrations (> 50 µg/mL), likely due to Hook effects, and upper detection limits differed substantially. Spike recovery ranged from 79 to 268%, with the most consistent results observed for sCAL turbo.

**Conclusions:**

Commercial assays reliably quantify serum calprotectin in the normal to moderately elevated range but are not interchangeable. Variability in detection limits and susceptibility to Hook effects may lead to underestimation in severe inflammation. Assay-specific reference ranges and additional dilution steps are recommended, particularly when extreme elevations are suspected.

**Supplementary Information:**

The online version contains supplementary material available at https://doi.org/10.1186/s40348-026-00267-8.

## Introduction

Calprotectin, a heterodimer of the S100A8 and S100A9 proteins (also termed MRP8/14), has emerged as a clinically relevant biomarker of innate immune activation [1]. It is released primarily by activated neutrophils and monocytes during inflammatory processes, and due to its stability and abundance, it can be reliably measured in blood and other body fluids [2].

While fecal calprotectin is widely established as a noninvasive marker for intestinal inflammation, measurement of calprotectin in serum is increasingly important for the assessment of systemic inflammatory conditions, particularly autoinflammatory diseases [3]. These disorders, such as Still’s disease (systemic juvenile idiopathic arthritis (SJIA) or adult-onset Still’s disease (AOSD)), periodic fever syndromes, and other monogenic or multifactorial autoinflammatory syndromes, are often difficult to distinguish from autoimmune or infectious diseases [4]. Commercially available serum calprotectin assays offer a potential tool to improve differential diagnosis and disease monitoring [5–7].

Several immunoassay platforms have been developed and commercialized to measure serum calprotectin, including enzyme-linked immunosorbent assays (ELISAs), chemiluminescent immunoassays (CLIAs), and particle-enhanced turbidimetric immunoassays (PETIAs) [8, 9]. These assays are designed for routine clinical laboratories and vary in sensitivity, dynamic range, and turnaround time. ELISA-based tests remain the most commonly used, offering high analytical sensitivity, though they require batch processing and are relatively time-consuming. Automated immunoassays, available from various diagnostic manufacturers, provide faster results with less manual handling, facilitating integration into routine diagnostic workflows [10, 11].

Despite the increased use of serum calprotectin (S100A8/A9 complex) in routine clinical settings, particularly in pediatric populations, some challenges remain. Inter-assay variability and the lack of universal standardization across different platforms applied in clinical routine laboratories can complicate interpretation and cross-study comparisons. Reference ranges are not yet harmonized, and cut-off values for distinguishing autoinflammatory conditions from other causes of systemic inflammation may differ depending on the assay used. Additionally, as calprotectin is not disease-specific, elevated values must be interpreted in the clinical context, ideally in combination with other biomarkers and/or genetic data.

In this study, we systematically compared commercially available assays for the measurement of serum calprotectin concentrations aiming for harmonization of assays and result reporting between laboratories.

## Methods

### Patients

We included serum from 40 patients with diagnosis of systemic juvenile idiopathic arthritis (SJIA, *n* = 10), Adult-onset Still’s disease (AOSD, *n* = 10), familial Mediterranean fever (FMF, *n* = 10), PAPA syndrome (*n* = 2), infection *n* = 5 and leukemia (*n* = 3) (Table 1). Patient samples were partly used already in the studies of Park et al. [6] and Foell et al. [8].

**Table 1:** Patient characteristics

	SJIA (*n* = 10)	AOSD (*n* = 10)	FMF (*n* = 10)	PAPA (*n* = 2)	Infections (*n* = 5)	Leukemia (*n* = 3)	Healthy controls (*n* = 10)
Age, mean(range), years	9.2 (2.4–14.3)	39.1 (23–63)	7.4 (2.3–17.0)	25.4 (16.7–34.2)	8.3 (0.1–15.6)	9.6 (8.4–12.1)	13.7 (10.2–16.9)
Sex: male/female, n	6/4	5/5	6/4	1/1	3/2	2/1	5/5
Percent of male/female	60/40	50/50	60/40	50/50	60/40	66/34	50/50
Leukocyte count, /µl (median + 95%CI)	13,401 (7100–22900)	14,158 (6530–32780)	10,355 (6500–14150)	9860 (6520–13200)	9165 (4300–12300)	1200 (n.a.)	5595 (5001–6490)
CRP, mg/dl	18.85 (9-15.7)	95.14 (16–190)	2.7 (0.03–5.5)	0.85 (0.5–1.2)	7.1 (1.7–13.8)	4.3 (2.1–6.4)	all negative
ESR, mm/h	66 (32–83)	49 (47–51)	43 (8–70)	24 (n.a.)	65 (35–96)	n.a.	n.a.

Data are expressed as mean ± 95% CI unless stated otherwise

*Abbreviations*: *SJIA* systemic juvenile idiopathic arthritis, *AOSD* adult-onset Still´s Disease, *FMF* familial mediterranean fever, *PAPA* pyoderma gangraenosum, acne and pyogenic arthritis syndrome, *CRP* C-reactive protein, *ESR* erythrocyte sedimentation rate

AOSD samples were provided by ImmunAID cohort (European Union’s Horizon 2020 research innovation program “ImmunAID”; H2020-EU.3.1.1.) (Medical Ethics Committee at the University of Muenster, Muenster, Germany Az 2020-476-f-S). PAPA syndrome samples were from our outpatient clinics (Medical Ethics Committee at the University of Muenster, Muenster, Germany Az 2015-670-f-S). All other samples were collected as previously reported. Those patients presented with fever of unknown origin and underwent a diagnostic checkup for differential diagnosis of SJIA [8].

Additionally, healthy pediatric controls (*n* = 10) were included – already measured for Park et al. [6] and Foell et al. [8]. These children underwent endocrinologic evaluation for growth velocity variations at the University Hospital Muenster, but were without signs of inflammation (supported by normal leucocyte count, negative CRP).

### Experimental setup

To evaluate the effects of exogenous proteins on serum calprotectin levels, human recombinant S100A8 (Kactusbio, Waltham, USA, Cat.no. KT-SA8-HE101-100UG), S100A9 (Kactusbio, Waltham, Cat.no. KT-SA9-HE101-100UG), the S100A8/A9 complex (Kactusbio, Waltham, Cat.no. KT-CAL-HE101-100UG), and a 45% S100A8 + 55% S100A9 mixture (based on molecular weight) were added to human serum (blood type AB, from Sigma-Aldrich, St. Louis, Missouri, USA, Cat.no. H4522) at final concentrations of 0.5, 1, 10, 50, and 100 µg/mL.

### Determination of calprotectin levels

**Fig. 1:**
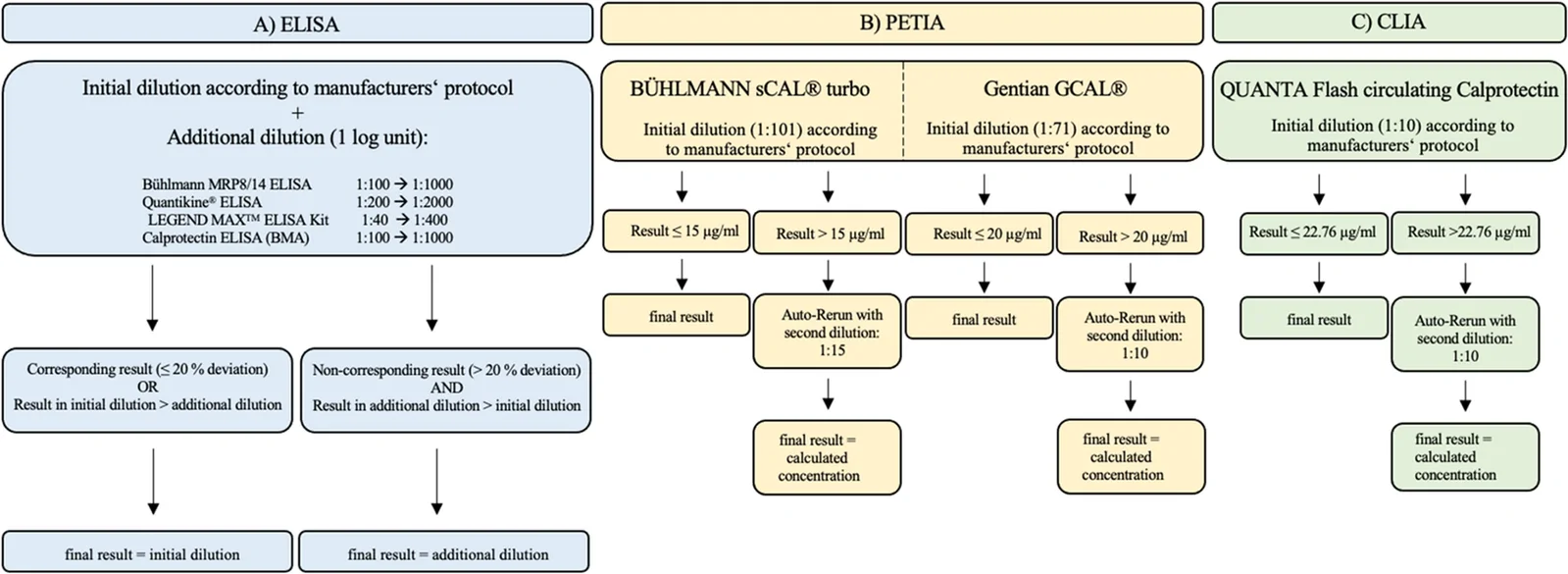
Applied work-flow in this study for different assays. **A** Initial dilution was chosen according to manufacturers‘ protocol: Bühlmann MRP8/14 ELISA: 1:100, Quantikine^®^ ELISA 1:200, LEGEND MAX™ ELISA Kit 1:40, Calprotectin ELISA (BMA) 1:100. A second dilution was perfomed (one log unit): Bühlmann MRP8/14 ELISA: 1:1000, Quantikine^®^ ELISA 1:2000, LEGEND MAX™ ELISA Kit 1:400, Calprotectin ELISA (BMA) 1:1000. If results are corresponding (≤ 20% deviation) or the result in initial dilution is higher than in additional dilution, this can be accepted as final result. In case of > 20% deviation and with higher results in additional diluation, the result of the additional dilution was defined as final result in this study. **B** For BÜHLMANN sCAL^®^ turbo initial dilution was 1:101. If result was ≤ 15 µg/ml, the result was taken as final result. If result of initial dilution was > 15 µg/ml another 1:15 dilution was applied automatically, resulting in an overall dilution of 1:1515. For Gentian GCAL^®^ initial dilution was 1:71. If result was ≤ 20 µg/ml, this was taken as final result. If result of initial dilution was > 20 µg/ml another 1:10 dilution was applied automatically, resulting in an overall dilution of 1:710. **C** For QUANTA Flash circulating Calprotectin initial diluation is 1:10. If result was ≤ 22.76 µg/ml, this was taken as final result. If result was > 22.76 µg/ml and Auto-Rerun-Option was activated, another 1:10 dilution was applied automatically, resulting in an overall dilution of 1:100

Calprotectin concentrations were quantified using four ELISA kits – BÜHLMANN MRP8/14, also referred to as BÜHLMANN sCAL^®^ ELISA (BÜHLMANN Laboratories, Switzerland referred to as “BHL EK”), Quantikine^®^ (R&D Systems, USA, “R&D”), LEGEND MAX™ (BioLegend, USA, “Biolegend”), and BMA Biomedicals MRP8/14 (Switzerland, “BMA”), two particle-enhanced turbidimetric immunoassays (PETIA): BÜHLMANN sCAL^®^ turbo (“sCAL”) and Gentian GCAL^®^ (Gentian Diagnostics, Norway, “GCAL”), and one chemiluminescent immunoassay (CLIA): QUANTA Flash Circulating Calprotectin (Werfen GmbH, Germany, “Werfen”), see supplemental Table 1 for details of assays. All assays were performed according to manufacturers’ protocols. For ELISAs, an additional dilution step (one log unit higher than the initial dilution) was applied to extend measurement range. In order to exclude high dose hook effect, samples have also been tested with the extended range on QUANTA Flash. In Fig. 1 we have summarized the applied work-flow in this study for the different assays.

### Assay principles and procedures

All ELISAs employ a sandwich format in which immobilized antibodies capture calprotectin from the sample, followed by binding of an enzyme-linked detection antibody. The enzyme-substrate reaction generates a color change proportional to calprotectin concentration, measured at 450 nm. Analyses followed the respective user manuals. See supplemental Table 1 for details of assays.

sCAL^®^ turbo and Gentian GCAL^®^ are particle enhanced turbidimetric assays (PETIA), that are run on automated clinical chemistry analyzers (Suppl. Table 1). The sCAL^®^ turbo and Gentian GCAL^®^ assays, performed on a Roche cobas^®^ c502 analyzer (University Hospital Münster, central laboratory), quantify calprotectin by immunoparticle agglutination. Light absorbance correlates with antigen concentration. For sCAL^®^ turbo, an automated secondary 1:15 dilution was applied when > 15 µg/mL (overall 1:1515). For Gentian GCAL^®^, a secondary 1:10 was applied when exceeding the upper limit (overall 1:710).

The QUANTA Flash Calprotectin assay (“Werfen”) is a chemiluminescent immunoassay (CLIA), performed on the BIO-FLASH^®^ system (University Hospital Bern, central laboratory), uses paramagnetic beads coated with calprotectin-specific antibodies and an isoluminol-conjugated detection antibody. The resulting chemiluminescent signal, proportional to calprotectin concentration, is automatically quantified. Initial dilution was 1:10, with an automated secondary 1:10 rerun for values > 22.76 µg/mL (overall 1:100). As with PETIA, the process is fully automated including dilution steps and calculation of results.

### Workflow, definition of reference levels and quality control

All assays were executed under standardized conditions following manufacturer protocols. ELISAs included an additional 10-fold dilution (see Fig. 1). ELISA measurements were performed in duplicate wells on each plate. Technical replicate variability was assessed from the duplicate optical density (OD) measurements and expressed as coefficient of variation (CV), calculated as SD/mean × 100. Measurements for which one or both duplicate readings exceeded the assay range were excluded from CV calculations. Spiked samples were additionally analyzed on separate assay plates at two different dilution levels (see Fig. 1) to assess dilutional behavior and potential high-dose effects.

Statistical analyses were performed using GraphPad Prism version 10 (GraphPad Software, Boston, MA, USA). Associations between calprotectin measurements obtained with different assays were assessed using Spearman’s rank correlation coefficient. Method comparison was further evaluated by Deming regression, using the extended-dilution BHL EK measurements as the comparator, to assess proportional and constant bias based on regression slopes and intercepts with 95% confidence intervals. Agreement between methods was assessed using Bland–Altman analysis after log10 transformation of calprotectin concentrations to account for the wide concentration range. Mean bias and 95% limits of agreement (mean difference ± 1.96 SD) were calculated. Only samples with quantitative results available for both methods were included in the respective pairwise analyses; missing or non-quantifiable out-of-range values were excluded.

Since serum is the only sample material that can be used for all assays (see supplemental Table 1), we decided to measure calprotectin in serum samples only. Assay-specific interferences (s. supplemental Table 3) were not available.

Since 2018, we use calprotectin measurement with the BHL EK ELISA system for scientific analyses (not routine clinical laboratory testing) [6, 8]. This assay is still our scientific reference assay and in the current study, we used the serum values obtained with serial dilutions into the linear assay range, measured by this BHL EK assay as comparator method (Ref. BHL EK), but was not considered a gold-standard due to a lack of assay standardization. Dilutions for extremely high values were up to 1:25.000.

## Results

### Detection of calprotectin in sera of healthy controls and in spiked serum

**Fig. 2:**
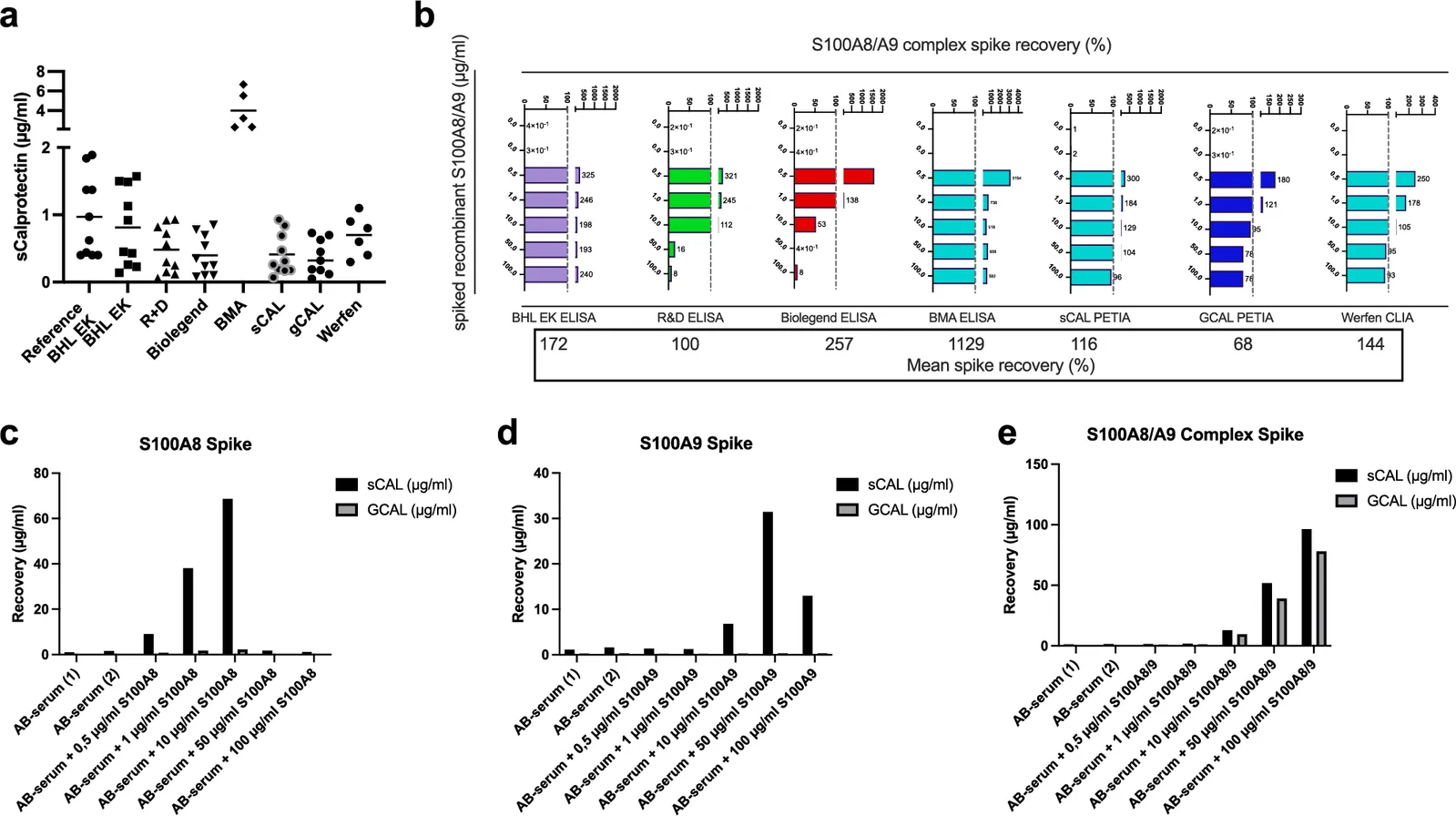
Detection of Calprotectin in sera of healthy controls and in spiked serum. **A** Calprotectin levels were measured in 10 healthy controls using the Reference ELISA (Bühlmann EK, plus four ELISAs (BHL EK, R + D, Biolegend, BMA), two PETIAs (sCAL turbo, gCAL), and one CLIA (Werfen). For BMA (5 individuals) and Werfen (6 individuals) measured values had to be reduced due to availability of serum. Median values in healthy controls varied substantially (0.29–6.67 µg/mL), with inter-assay differences exceeding one log scale. Especially the BMA-assay calculated much higher levels. **B** Spike recovery of commercially purchased Calprotectin complex was tested in normal serum (AB serum) in all assays. Spike recovery was calculated after subtraction of the endogenous calprotectin concentration measured in the corresponding unspiked serum. Mean recovery ranged from 68 to 1129%. With sCAL turbo showing the most consistent recovery (116%) across the spectrum of values (very low to very high). **C** Spike recovery of S100A8 measured in sCAL and gCAL. **D** Spike recovery of S100A9 measured in sCAL and gCAL. **E** Spike recovery of S100A8 plus S100A9 measured in sCAL and gCAL

To assess calprotectin levels of healthy controls, 10 healthy children were measured in all assays. Median values varied substantially (0.29–3.2 µg/mL), with inter-assay differences exceeding one log scale. BHL EK, sCAL, GCAL and Werfen measured in the range of normal controls given by the manufacturers, while Biolegend and R&D gave values in part below the range and BMA far above the range for normal controls (see Table 2; Fig. 2a).

**Table 2:** Serum calprotectin levels (µg/ml) determined by different assays

Assay	SJIA (median + 95% CI) *n* = 10	AOSD (median + 95% CI) *n* = 10	FMF (median + 95% CI) *n* = 10	Infections (median + 95% CI) *n* = 5	Leukemia (median + 95% CI) *n* = 3	Healthy controls (*n* = 10) Median (Min-Max)	HC range due to Manufacturer
Reference BHL EK	67 (19–200)	18 (8-150)	5 (1.7–15)	16 (3–35)	1.5 (1.4-4)	0,8 (0,4 − 1,9)	1,14 (+/- 2,9)
BHL EK	73 (13–176)	17 (8-123)	5 (2–15)	16 (3–32)	1.8 (1.5-4)	0,69 (0,14 − 1,6)	1,14 (+/- 2,9)
R + D	29 (9–44)	10 (5–77)	2 (0.7-6)	8 (1.4–16)	0.4 (0.06–1.6)	0,43 (0,06 − 0,93)	0,48 − 6,5 (range)
Biolegend	29 (9–68)	8 (5–58)	2 (0.7-6)	7 (1.4–14)	0.2 (0.06–1.7)	0,3 (0,08 − 0,85)	0,11 − 3,52 (range)
BMA	205 (41–310)	65 (14–428)	34 (15–42)	37 (16–114)	11 (n.a.)	3,2 (2,3–6,7)	0,5 − 3,5 (range)
sCAL	30 (8–84)	9 (5–60)	3 (1.1-8)	8 (1.8–15)	1.0 (0.7–1.8)	0,29 (0,07 − 0,93)	0,24 − 2,01 (95% CI)
gCAL	30 (7–83)	7 (3–55)	3 (0.9-7)	7 (0.8–14)	0.6 (0.4–1.3)	0,32 (0,05 − 0,73)	< 1,75
Werfen	32 (9–47)	14 (4-122)	3 (1.9-9)	11 (5–16)	3 (n.a.)	0,7 (0,3 − 1,1)	< 2,0

*Abbreviations*: *SJIA* systemic juvenile idiopathic arthritis, *AOSD* adult-onset Still´s Disease, *FMF* familial mediterranean fever, *HC* healthy controls

Spike recovery ranged from 79 to 268%, with sCAL turbo showing the most consistent recovery (116%) across all concentration ranges, when S100A8/A9 complex was added (Fig. 2b). All assays overinterpreted values in low concentration ranges. Measured concentrations ranged between 180% (GCAL) and 3154% (BMA) for 0,5 µg/ml and between 138% (Biolegend) and 736% (BMA) for 1,0 µg/ml. For concentrations in a moderate range (10 µg/ml) most assays had similar measurements, except BMA with exaggerating up to 518%. Technical replicate variability of the ELISA measurements was low, with median duplicate-well CVs of 1.9% for BHL EK, 1.4% for R&D, and 1.1% for BioLegend. BMA showed somewhat higher variability (median CV 4.8%), although this remained small relative to the pronounced differences observed in spike recovery (see suppl. Table 2). Serial dilution revealed additional assay-dependent effects. BHL EK showed consistent back-calculated concentrations across dilution levels, whereas further dilution increased the measured concentration for R&D and BioLegend at higher spike concentrations; for BioLegend, the measured concentration at the 10 µg/mL S100A8/A9 spike increased from 5.27 to 9.76 µg/mL after tenfold further dilution. These findings support concentration-dependent assay interference and are consistent with a suspected high-dose effect in selected samples. High levels (50 to 100 µg/ml) were nearly optimally measured by sCAL and Werfen, while GCAL and BHL EK showed still acceptable catch-up.

Single spiked proteins (S100A8 or S100A9; Fig. 2c, d) were interestingly only measured in sCAL assay, but for S100A8 not reliably. Adding single proteins (S100A8 and S100A9) together was instead reliably measured by GCAL and sCAL (Fig. 2e), but not in the other assays.

### Assay comparability in patients with autoinflammation

**Fig. 3:**
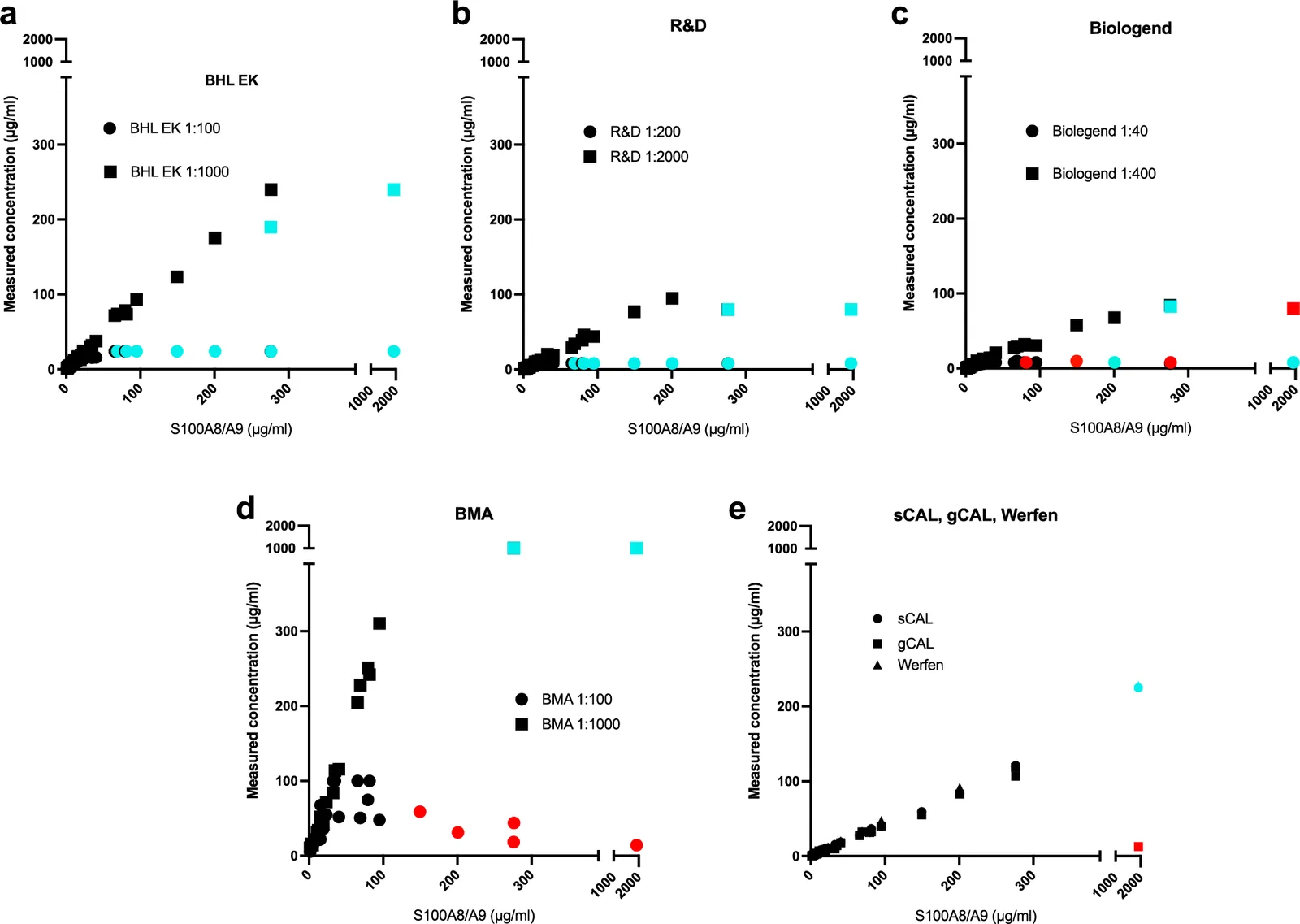
Assay comparability in patients with Autoinflammation. Calprotectin levels were analysed in 40 patients with systemic juvenile idiopathic arthritis (sJIA, *n* = 10), adult-onset Still’s disease (AOSD, *n* = 10), familial Mediterranean fever (FMF, *n* = 10), PAPA syndrome (*n* = 2), infections (*n* = 5), leukemia (*n* = 3), in (**A**) BHL EK, (**B**) R + D, (**C**) Biolegend, (**D**) BMA, and (**E**) in 2 PETIA (sCAL and gCAL) and 1 CLIA (Werfen). Values obtained with the extended-dilution BHL EK assay are shown as comparator values on the x-axis. Light blue dots indicate samples correctly identified by the respective assay as exceeding its upper detection limit (> ULD). Red dots indicate false-low results, defined as apparently quantitative values that substantially underestimated the comparator concentration and were not flagged as > ULD by the assay. Such false-low results may arise from analytical interference, including a high-dose hook effect

To test the assays in monogenic and multifactorial autoinflammatory conditions against controls, we analyzed serum from 40 patients, partly published before (Fig. 3).

As several samples were known to contain extremely high calprotectin concentrations (up to approximately 2,000 µg/mL), an important question was whether assays correctly identified concentrations exceeding their upper detection limit (> ULD) or instead reported spuriously low quantitative results (false-low; see supplemental Table 3 for definitions). The ability to indicate false low values is important for routine laboratory measurements, to avoid too many cost-intensive and time-consuming routine dilution steps. We therefore added patient samples with values above 50 µg/ml (5 samples between 50 and 100 µg/ml and 5 samples above 100 µg/ml) and investigated the comparability of assays especially in these samples with extremely elevated calprotectin levels that are typical of certain autoinflammatory conditions.

The comparability of tested ELISA assays was clearly variable. BHL EK correctly identified all samples above 50 µg/mL as > ULD at the initial dilution (light blue circles Fig. 3). Two of the highest concentrations measured with the second dilution were also indexed correctly (> ULD). R&D was similar in correctly determining values to be > ULD but showed a plateau of values above 100 µg/ml. Biolegend reported false low results in 3 of 10 samples above 50 µg/ml for the 1:40 dilution and was not able to measure values above 82 µg/ml in the 1:400 dilution, giving only an average of 32% (range 25% − 39%) recovery of calprotectin levels compared to BHL reference. Only one sample measured at 1:400 dilution was correctly indicated as > ULD. BMA reported false low results in 5 of 10 samples above 50 µg/ml for the 1:100 dilution. In the 1:1000 dilution instead the assay exaggerated calprotectin levels giving values up to 22-times higher than with its 1:100 dilution.

PETIA and chemiluminescence assays showed a high comparability with a very good linearity compared to BHL reference and almost identical values. For the highest sample (1911 µg/ml in a PAPA patient) sCAL and Werfen correctly indicated > ULD, while GCAL indicated this value as false low consistent with a high-dose hook effect.

Maximum measurable concentrations were limited in BHL EK (190 µg/ml), R&D (95 µg/mL), Biolegend (82 µg/mL), GCAL (118 µg/mL), and Werfen (227 µg/mL), whereas only the BMA ELISA measured up to 1000 µg/mL. sCAL turbo demonstrated partial capacity to estimate high values, albeit with underestimation.

While assays showed strong and significant (*p*<0,0001) correlation with the Ref. BHL EK ELISA (r >/= 0,96) except BMA (*r* = 0.52; *p* = 0,01), Deming regression (for ELISA assays 10-fold dilution was used; out-of-range and missing values were excluded) revealed substantial assay-dependent proportional bias relative to the extended-dilution Ref. BHL EK. While the BHL EK dilution showed no significant proportional or constant bias, slopes differed markedly from unity for R&D (0.49, 95% CI 0.45–0.53), BioLegend (0.31, 95% CI 0.28–0.35), BMA (3.51, 95% CI 3.06–3.97), sCAL (0.43, 95% CI 0.41–0.44), and Werfen (0.44, 95% CI 0.42–0.46), demonstrating substantial proportional bias despite strong correlations between assays. Including the false low value GCAL showed no meaningful linear agreement in the complete dataset. Excluding this value, GCAL (0.41, 95% CI 0,3727 to 0,4389) was in the same range like sCAL and Werfen.

Bland–Altman analysis revealed assay-dependent differences in agreement with the extended-dilution Ref. BHL EK (Supplementary Figure S1). Repeated BHL EK measurements showed minimal overall bias and comparatively narrow limits of agreement, supporting good agreement between dilution steps. In contrast, the other assays showed systematic positive or negative biases relative to BHL EK, with several assays additionally displaying concentration-dependent differences. This was particularly evident for sCAL and Werfen, for which differences increased with increasing calprotectin concentrations. Thus, despite the strong correlations observed between most assays, Bland–Altman analysis demonstrated systematic and, for some assays, concentration-dependent disagreement, supporting limited interchangeability of absolute calprotectin concentrations across platforms.

Direct comparison of the automated assays showed the closest agreement between sCAL and Werfen (bias − 0.038 log10 units; 95% limits of agreement [LoA] − 0.129 to 0.054), corresponding to a mean ratio of 0.92 (LoA 0.74–1.13). Comparisons involving GCAL showed greater differences, with biases of − 0.081 for sCAL versus GCAL and + 0.086 log10 units for GCAL versus Werfen. Thus, sCAL and Werfen demonstrated the closest numerical agreement among the automated assays (figure S1).

### Comparability of patient levels with previous studies and between assays

In former studies, we already published calprotectin levels for different diseases and distinct activity states [2, 6, 8, 12–15]. As most of the patient samples were already used in the study by Park et al. [6] and Foell et al. [8] we could directly compare levels of the Ref. BHL EK with published levels and confirmed a high reproducibility with a mean catch-up percentage of 98% (+/- 15 SEM), see figure S2. Correlation between both assays was *r* = 0.99 (*p* < 0.0001).

Comparing patient levels (especially SJIA, AOSD, FMF, infections) between assays (Table 2) revealed rather similar median levels for R&D, Biolegend, sCAL, GCAL and Werfen, while BHL EK consistently measures calprotectin levels 2.0–2.5 times higher than the other assays. Instead, the BMA assay is measuring calprotectin levels 3–6 times higher than BHL EK, and 6–12 times higher than the other assays.

### Recommended workflow of serum calprotectin measurement and interpretation

**Fig. 4:**
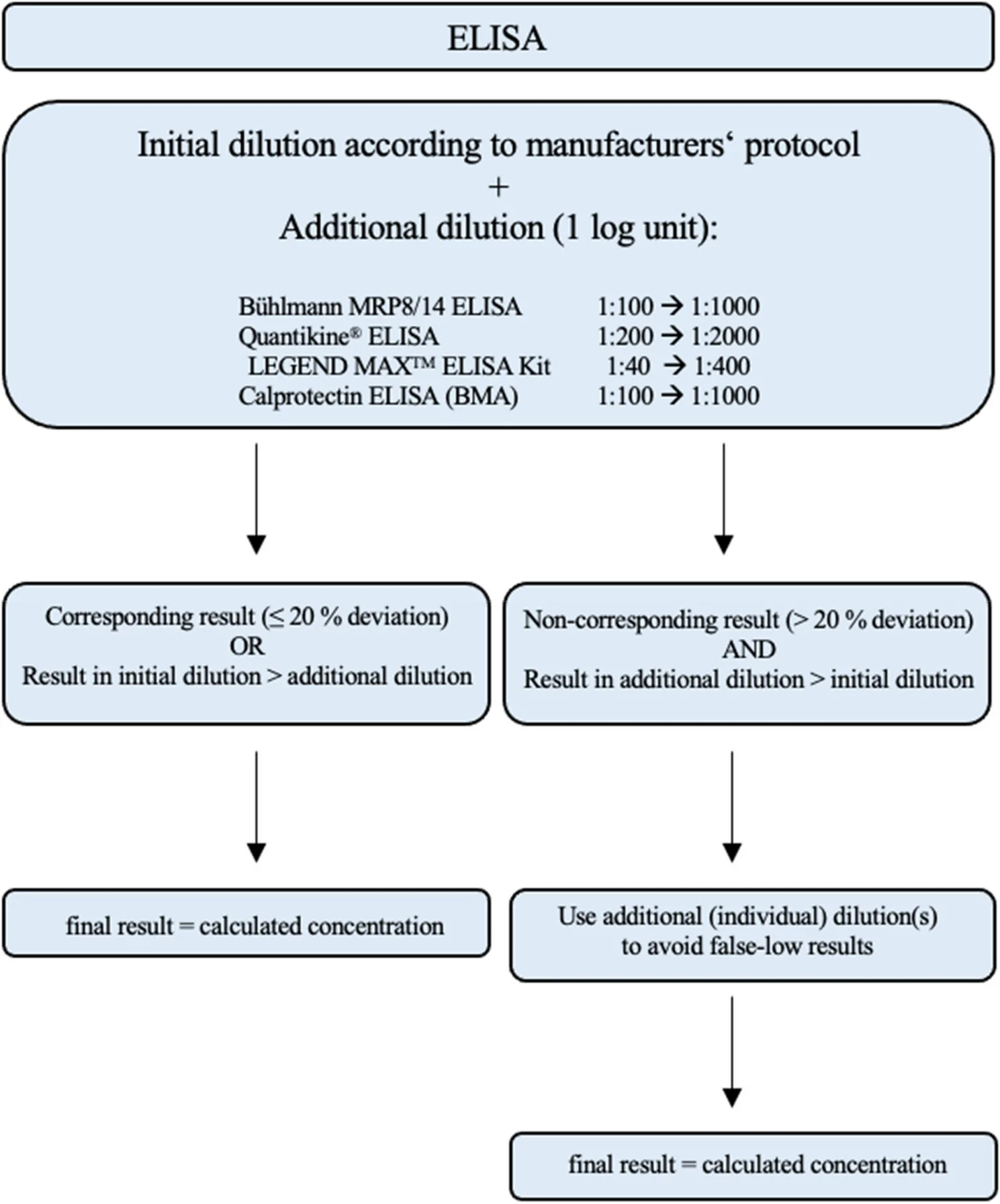
Recommended workflow of serum calprotectin measurement in ELISA assays. For ELISA assays, we suggest the dilution and evaluation as depicted. For exact results, another third (individual) dilution is recommended, if extremely high levels are expected or indicated by the assay

For ELISA methods, we suggest the following dilution strategy (Fig. 4): application of an initial dilution according to manufacturer‘s protocol plus an additional dilution, that is 1 log unit higher. If results are corresponding (≤ 20% deviation) or the result in initial dilution is higher than in additional dilution, this can be accepted as final result. If results are non-corresponding (> 20% deviation) and with higher results in additional dilution than in the initial one, an additional dilution(s) should be carried out to avoid false-low results. Dilutions should be taken into account to calculate the final result. Recommendation for troubleshooting and refinement for measurement of serum calprotectin in human samples according to used assay are summarized in supplemental Table 3.

## Discussion

In our study, we systematically compared commercially available assays for serum calprotectin with an internal comparator method and could test the validity of these assays in the context of autoinflammation. With our approach, we can show, which pitfalls arise from certain assays and how to carefully interpret especially biosamples with extremely elevated serum levels of calprotectin.

A small healthy control cohort was measured by BHL EK, sCAL, GCAL and Werfen reliably in a comparable range and according to manufacturer´s given range for Healthy controls. Testing spiked levels of calprotectin reveals, that all assays overinterpreted values in low concentration ranges, while only sCAL and Werfen were nearly optimally measuring extremely high levels between 50 µg/ml and 100 µg/ml. Individual protein levels of the heterocomplex calprotectin should ideally not be measured by the assays, as according to manufacturer, assays detect the heterocomplex (calprotectin). Nevertheless, in high concentrations single protein levels were measured with sCAL, indicating some cross-reactivity.

Patient levels have to be interpreted according to applied assay, because absolute values are not per se interchangeable. But with some caution an interassay comparability is given for R&D, Biolegend, GCAL, sCAL and Werfen. Comparing the disease cohorts for SJIA, AOSD and FMF, as prototypical monogenic and multifactorial autoinflammatory diseases, our analysis shows comparable range intervals for these assays, but values with BHL EK have to multiplied by a factor of 2.0-2.5, as reported elsewhere [8]. The only assay delivering a value range not comparable to the other assays, is BMA.

Looking at accuracy, both Biolegend and BMA are having a problem to show correctly too high values, probably due to a Hook-effect. This effect occurs in ELISA assays, when extremely high concentrations of analyte saturate both the capture and detection antibodies, preventing proper “sandwich” complex formation. This results in a false low signal and underestimation of the true analyte concentration. Both assays would miss to inform treating physicians in up to 30% (Biolegend) or 50% (BMA) of cases with extremely elevated calprotectin, like FMF or Still syndrome or PAPA. Especially in these cases, calprotectin levels have a high diagnostic impact and patients are at risk to be missed. The BMA assay is additionally exaggerating values until 100 µg/ml. When comparing patient values obtained with BHL EK in our current study to historical values reported in previous publications, we confirm very good reproducibility and comparability.

We and others have shown in the past that measurement of calprotectin in serum of patients with autoimmune and autoinflammatory conditions is helpful for diagnosing and monitoring of these patients [2–4, 6, 8, 9, 12–18]. Over the past years, the technology readiness level of this biomarker has advanced through successive publications, reaching level 7—“operating in an operational environment at pre-commercial scale”—as comprehensively described by Pardeo et al. [19]. At our own institution routine measurement of the biomarker is provided in the central laboratory already for several years. Validation of the sCAL turbo assay on the automated instrument Roche cobas^®^ c502 analyzer has led to daily clinical routine measurements with random access and provides for the patient care of our and many other physicians in Germany important biomarker information [8]. The automated PETIA and CLIA platforms generally showed consistent performance across the clinically relevant concentration range and offer practical advantages for implementation in routine laboratory testing. However, method-comparison and agreement analyses revealed systematic assay-dependent differences in absolute calprotectin concentrations, and individual analytical limitations were observed at very high concentrations. Thus, results obtained with different platforms should not be considered directly interchangeable. For the GCAL assay close feedback with the clinicians sending the samples seem to be necessary, to not miss false low measured values, if senders are expecting potentially extremely elevated serum levels. A false-low measured value in a patient with severe autoinflammation (in our study a PAPA patient) might cause delayed diagnosis or exclusion of severe autoinflammatory disease. In cases of high clinical suspicion, unexpected low results should therefore prompt additional dilutions in al assays. Especially in ELISA assays, we therefore recommend – if assays are used for clinical routine – to introduce an additional dilution step 1 log unit higher, as depicted in Fig. 4. Even though the two random access tests, sCAL and Werfen, have limited upper level of detection, these tests did not miss any samples with very high levels of calprotectin, but of course implementation can vary depending on laboratory workflow and available automation platforms.

Importantly, serum calprotectin measurement provides advantages over classical acute-phase reactants such as C-reactive protein (CRP) and erythrocyte sedimentation rate (ESR). Unlike CRP, which is hepatically synthesized in response to interleukin-6, calprotectin directly reflects myeloid cell activation and may rise earlier or remain elevated in conditions where CRP is less discriminatory [3, 20]. This distinction can be clinically useful in distinguishing autoinflammatory from autoimmune or infectious etiologies, particularly in complex pediatric or adult cases with recurrent fever of unknown origin. Furthermore, serial measurements using commercially available assays have shown potential for monitoring treatment response, especially to biologics targeting interleukin-1 or interleukin-6 pathways [12, 15, 21, 22]. The development and implementation of internationally accepted calibration standards may facilitate harmonization of calprotectin measurements and improve inter-laboratory comparability, although assay- and platform-specific analytical differences may persist despite standardized calibration [23].

Limitations of our study are incompleteness of all available calprotectin assays on the market, and a limitation of patient samples, that resulted in fewer sample analysis for BMA and Werfen. Another limitation is the heterogenous study population, that was necessary to analyze calprotectin levels across the whole concentration range, but results in small sizes of several diagnostic subgroups. This limits the generalizability of disease-specific conclusions and we cannot provide certain cut-off levels for the diagnosis of SJIA, AOSD or FMF, as in earlier studies.

In conclusion, commercially available serum calprotectin assays represent a valuable addition to the diagnostic toolbox for autoinflammatory diseases. Their ability to capture innate immune activation provides improved differentiation from autoimmune and infectious conditions and offers opportunities for disease monitoring and therapeutic guidance. Nevertheless, assay-specific reference ranges should be taken into account and additional dilution steps are recommended, if extreme elevations are suspected. Ongoing efforts toward assay harmonization, establishment of validated disease-specific cut-offs, and integration into diagnostic algorithms will further enhance its clinical utility.

## Supplementary Information

Supplementary Material 1.

## Data Availability

All data relevant to the study are included in the article.
