# Supplementary material for: Challenges in measuring serum calprotectin in autoinflammation: a comparative study of different immunoassays
Source: Mol Cell Pediatr. 2026 Nov 10;13:54. doi: 10.1186/s40348-026-00267-8 (PMC13748079; doi:10.1186/s40348-026-00267-8)
Supplement: Supplementary file 1 [file 40348_2026_267_MOESM1_ESM.docx]

**Challenges in Measuring Serum Calprotectin in Autoinflammation: A Comparative Study of different Immunoassays**

Helmut Wittkowski^1^, Carolin Park^1^, Melanie Saers^1^, Sabrina Fühner^1^, Susanne Schleifenbaum^1^, Claas Hinze^1^, Manfred Fobker^2^, Andrea Karolin^3^, Michael P. Horn^3^, Bernhard Schlüter^2^, Dirk Foell^1^

^1^University Children’s Hospital Muenster, Department of Pediatric Rheumatology and Immunology, Muenster, Germany

^2^University Hospital Muenster, Central Laboratory, Muenster, Germany

^3^University Institute of Clinical Chemistry, University Hospital of Bern, Bern, Switzerland

Corresponding author details:

Prof. Dr. med. Helmut Wittkowski

University Children’s Hospital Muenster, Department of Pediatric Rheumatology and Immunology

Albert-Schweitzer-Campus 1, W30

48161 Muenster

Germany

E-Mail: [helmut.wittkowski@ukmuenster.de](mailto:helmut.wittkowski@ukmuenster.de)

| General information | Technique | ELISA |  |  |  | PETIA |  | CLIA |
| --- | --- | --- | --- | --- | --- | --- | --- | --- |
|  | Manufacturer | BÜHLMANN Laboratories AG Schoenenbuch, Switzerland | R&D systems, Inc. Minneapolis, USA | BioLegend Inc. San Diego, California, USA | BMA Biomedicals Augst, Switzerland | BÜHLMANN Laboratories AG Schoenenbuch, Switzerland | Gentian Diagnostics Moss, Norway | Werfen GmbH Munich, Germany |
|  | Name (order number) | BÜHLMANN MRP8/14 ELISA (EK-MRP8/14) | Quantikine^®^ ELISA (DS8900) | LEGEND MAX^TM^ ELISA Kit (439707) | Calprotectin (MRP8/14) ELISA (S-1011) | BÜHLMANN sCAL® turbo (B-KSCAL-RSET, B-KSCAL-CASET, B-KSCAL-CONSET) | Gentian GCAL^®^ (1201, 1219, 1251) | QUANTA Flash circulating Calprotectin (PN 066701365) |
|  | User manual | [https://www.buhlmannlabs.ch//wp-content/uploads/2015/03/ek-mrp8-14_ifu_ce_12-11-13_.pdf](https://www.buhlmannlabs.ch/wp-content/uploads/2015/03/ek-mrp8-14_ifu_ce_12-11-13_.pdf) | <https://resources.rndsystems.com/pdfs/datasheets/ds8900.pdf?v=20250423> | <https://d1spbj2x7qk4bg.cloudfront.net/Files/Images/media_assets/pro_detail/datasheets/439707_R06.pdf?v=20250407123848> | <https://www.bma.ch/files/product/S-1011-Lot-33.pdf> | <https://www.buhlmannlabs.ch/wp-content/uploads/2015/01/B-KSCAL-RSET_IFU-CE_VA1.1-2023-11-15_EN.pdf> | <https://6752890.fs1.hubspotusercontent-na1.net/hubfs/6752890/IFU%20IVDR%20-%20GCAL/1201-1202%20Reagent%20Kit/1201-1202%20IFU%20Gentian%20GCAL%20Reagent%20Kit%207704-v06en.pdf> | [https://www.werfen.com/na/en/quanta-flashr-circulating-calprotectin-reagents (Log-in requiered)](https://www.werfen.com/na/en/quanta-flashr-circulating-calprotectin-reagents%20(Log-in%20requiered)) |
|  | Application | In-vitro diagnostic | Research only | Research only | Research only | In-vitro diagnostic | In-vitro diagnostic | In-vitro diagnostic |
|  | IVDR certified | no | no | no | no | no | yes | yes |
|  | Samples per plate | 40 | 40 | 40 | 43 | individual measurements | individual measurements | individual measurements |
|  | Compatibility to clinical chemistry analysers | - | - | - | - | Abbot (Alinity, Architect c-series) Beckman Coulter (AU Series: AU480) Mindray (BS-480) Ortho (Vitros 7600) Roche (cobas c303, c501/c502, c503) Siemens (Atellica CH 930) Thermo Fisher Scientific (Indiko/Indiko Plus) The Binding Site (Optolite) | Abbott Laboratories (Architect, Alinity) Beckman Coulter (AU) Binding Site (Optilite) Mindray (BS) Roche (Cobas) Siemens (Atellica) | BIO-FLASH®: fully automated, random access chemiluminescent analyzer |

**Supplemental table S1:** Comparison of assays for MRP8/14 measurement

| Specimen and sample handling | Specimen collection | Serum: Use plain tubes, mix, leave to clot for 45 min. at room temperatur protected from ligth; centrifuge at 1800 x g for 15 min. at room temperature, remove serum immediately Plasma: Use EDTA tubes, centrifuge at 3000 x g for 5 min. at room temperature within 4 h of collection | Serum: Use serum separator tube, leave to clot for 30 min. at room temperatur; centrifuge at 1000 x g for 15 min., remove serum immediately Plasma: Use EDTA og heparin tubes, centrifuge at 1000 x g for 15 min. within 30 min. of collection | Serum: Use a serum seperator tube, leave to clot for 30 min., centrifuge at 1000 x g for 10 min., remove serum immediately Plasma: Use citrate, heparin or EDTA tubes, centrifuge at 1000 x g for 10 min. within 30 min of collection | no information available | Serum: use tubes without additives or gel seperator tubes, centrifuge at 1800 x g for 15 min. at room temperature, transfer into plain tubes within 6 h after collection | Serum: Non-gel tubes recommended, leave to clot for 30 min., centrifuge within 2 h after collection Li-Heparin plasma: centrifuge within 2 h after collection | Serum, EDTA plasma: centrifugate at 3000 x g for 30 min. within 3 h of collection, remove serum immediately Citrate plasma: centrifugate at 3000 x g for 30 min. within 24 h of collection |
| --- | --- | --- | --- | --- | --- | --- | --- | --- |
|  | Sample storage after centrifu-gation | Serum/plasma: 2-8°C for up to 30 days; ≤ -20°C for up to 6 months | Serum/plasma: store immediately at ≤ -20°C avoid repeated freeze-thaw cycles | Serum/plasma: store immediately at < -70°C avoid repeated freeze-thaw cycles | Biological fluids: ≤ -20°C use aliquot only once | Serum: ≤ 28°C for up to 15 days 2-8°C for up to 16 days ≤ -20°C (stable for at least 1 month) ≥ 6 freeze-thaw cycles not recommended | Serum/heparin plasma: 2-8°C for up to 48 h | Serum/plasma: room temperature for up to 48 h 2-8°C for up to 14 days (serum, citrate plasma) 2-8°C for up to 7 days (EDTA plasma) ≤ - 20°C for > 14 days 3 freeze-thaw cycles allowed |
|  | Sample volume | 10 µl | 10 µl | 10 µl | 10 µl | 100 µl | 100 µl | 100 µl |
| Range and dulition procedure | Standard range | 4 - 240 ng/ml | 0.625 - 40 ng/ml | 3.13 - 200 ng/ml | 15.63 - 1000 ng/ml | Measuring range: 0,23-15 µg/ml (extende 0.23-225 µg/ml) | Measuring range: 0,4-20.3 mg/l (security zone 95 mg/l); depending on calibrator an chemical analyzer | Measuring range: 0.18 - 22.76 µg/ml (extended 0.18-227.6 µg/ml) Cut-off = 2 µg/ml |
|  | Standard dilution (material) | 1:100 (human serum, human plasma) | 1:200 (serum) 1:100 (plasma) additional use: cell culture supernates, tissue lysates, saliva, urine, human milk, fecal extract | 1:40 (serum, plasma) additional use: cell culture supernatant, urine, saliva, other biological fluids | 1:100 (serum, plasma) | Cobas c502 (Roche): 1:101 (1.6 µl serum + 120 µl reagent R1 + 40 µl reagent R2) | Cobas c502 (Roche): 1:71 (3 µl serum + 180 µl reagent R1 + 30 µl reagent R3) | 1:10 |
|  | Dilution procedure | Use further dilution if initial concentration reads greater than the highest calibrator | Use further dilution if values are higher than the highest standard | Unknown samples should be run at a number of diliations; use higher/lower dilution, if a sample's absorbance falls outside the linear portion of the standard curve | Adapt dilution to be in the linear part of the standard curve | Cobas c502 (Roche): Automated second dilution if result is > 15 µg/ml (1:15) | Cobas c502 (Roche): Automated second dilution if result is >20 µg/ml (1:10) | Auto-Re-Run option with second dilution (1:10) when result is > 22,76 μg/ml |

| Technical information | Information on technique | Selective measurement of MRP8/14-antigen by sandwich ELISA | Quantitative determination of human S100A8/S100A9 heterodimer concentrations by sandwich ELISA | Quantitation of human MRP8/14 by sandwich ELISA | Specific determination of calprotectin in biological fluids by sandwich ELISA | Turbidimetric assay for quantitative determination of calprotectin in human serum samples on automated clinical analyzers | Turbidimetric aassay for quantitative measurement of calprotectin in human plasma and serum on automated clinical analyzers | Quantitative determination of Calprotectin/S100A8/A9 in human serum and plasma samples by sandwich-immunoassay |
| --- | --- | --- | --- | --- | --- | --- | --- | --- |
|  | Capture antibody | Monoclonal, specific to the MRP8/14 heterodimeric and polymeric complexes^1^ | Monoclonal, specific for human S100A8/S100A9 Heterodimer | Anti-Human MRP8/14 capture antibody | Highly specific, monoclonal antibody | Beads coated with polyclonal antibody against human calprotectin | Beads coated with avian antibody against human calprotectin | Beads coated with calprotectin specific capture antibody |
|  | Detection antibody | Monoclonal, no further information | Monoclonal, specific for human S100A8/S100A9 Heterodimer | Human MRP8/14 detection antibody | Polyclonal, chicken antibody | not necessary in PETIA | not necessary in PETIA | Isoluminol-conjugated anti-calprotectin antibody |
| Results | Calculation of results | Standard cuve must be generated for each set of samples assayed | Standard curve must be generated,  use of computer software is recommended | Standard curve must be generated, use of computer software is recommended | Standard curve must be generated, use computer software or manually | Results are automatically calculated on clinical chemistry analyzer | Results are automatically calculated on clinical chemistry analyzer | Results are automatically calculated, depending on lot-specific master curve (loaded via scan code) |
|  | Normal values [further information] | Serum: 1.14 µg/ml (median), 2.9 µg/ml (95th percentile) EDTA plasma: 0.79 µg/ml (median), 2.7 µg/ml (95^th^ percentile) [apparently healthy and asymptomatic adult blood donors; clinical relevant cut-off has to be established for each indication by each laboratory] | Serum: 481- 6540 ng/ml (range) EDTA plasma: 127 - 1395 ng/ml (range) Heparin plasma: 298 - 1640 ng/ml (range) [Apparently healthy volunteers, no medical history available] | Normal human serum and plasma: 0.11 - 3.52 µg/ml (range) [no further information] | Serum/plasma: 500 - 3500 ng/ml [no further information] | Serum (native tube):  0.24-2.01 µg/ml (95% CI) Serum (gel seperator): 0.19-1.8 µg/ml (95% CI) [adult, self-declared healtyh individuals; according to CLSI C28-A3] | Upper reference limit: Serum without gel: < 1.41 mg/l Serum with gel: < 1,75 mg/l Li-Hep plasma without gel: < 0.97 mg/l Li-Hep plasma with gel: < 1.69 mg/l [ostensibly healthy adults; every laboratory should determine a local reference limit] | Serum/plasma: < 2.0 µg/ml (95th percentile) [apparently healthy blood donors] |

Information of assays for MRP8/14 measurement in human blood. Unless stated otherwise, all information is according to the manufacturer. MRP8/14: Myeloid-related protein 8/14; ELISA: Enzyme linked immunosorbent assay; PETIA: particle-enhanced immuno-turbidimetric assay; CLIA: chemiluminescent immunoassay technology; IVDR: In vitro diagnostic regulation; IVD: in vitro diagnostic medical device; CLSI: Clinical and laboratory standards institute

^1^Hessian PA, Fisher L. The heterodimeric complex of MRP-8 (S100A8) and MRP-14 (S100A9). Antibody recognition, epitope definition and the implications for structure. Eur J Biochem. 2001 Jan;268(2):353-63. doi: 10.1046/j.1432-1033.2001.01894.x. PMID: 11168370.

**Supplemental table S2:**

Technical replicate variability in with Calprotectin complex (S100A8/A9) spiked serum samples. Technical replicates were available in the 4 ELISA assays.

| **Assay** | **Evaluable duplicate pairs** | **Mean CV^1^ (%)** | **Median CV^1^ (%)** | **Range (%)** |
| --- | --- | --- | --- | --- |
| BHL EK | 10,00 | 2,16 | 1,88 | 0.12–4.35 |
| R&D | 6^2^ | 2,30 | 1,38 | 1.08–5.89 |
| BioLegend | 10,00 | 1,70 | 1,13 | 0–4.87 |
| BMA | 10,00 | 6,25 | 4,83 | 1.40–13.02 |

^1^CV = Coefficient of Variation

^2^For R&D, conditions in which one or both OD readings were reported as “OVER” could not be used for calculation of duplicate CV.

**Supplemental table S3:**

Technical terms and their description.

| **Technical Term** | **Description** |
| --- | --- |
| False low | Assay indicates value as low, but true analyte values are by far higher = analytical artifact due to assay interference or high-dose hook effect |
| True low | Assay indicates value as low and true analyte value is low |
| > ULD =  > Upper limit of detection | > Assay specific limit of detection = true values are above the linear range of the assay curve; assay indicates values as “too high” |
| < LLD =  < Lower limit of detection | < Assay specific limit of detection = true values are below the linear range of the assay curve; assay indicates values as “too low” |
| Hook effect | extremely high concentrations of analyte result in an incorrectly low signal and underestimation of the true analyte concentration |

| Preanalytical phase | Sample collection: wrong blood collection tube |
| --- | --- |
|  | Assay specific interfering substances: |
|  | Bühlmann MRP8/14 ELISA: hemolytic, lipaemic, icteric samples; no information on drug interference Quantikine® ELISA: possibility of interference cannot be excluded, no further specific information LEGEND MAX^TM^ ELISA Kit: hemolytic samples; no further information on interfering substances Calprotectin (MRP8/14) ELISA (BMA): no information on interfering substances BÜHLMANN sCAL® turbo: hemolytic, lypolytic samples; Rheumatoid factor >88.3 U/ml; severel drug intereferences tested negative Gentian GCAL®: no interference to Rheumatoid factor (due to avian antibody), no interference with haemoglobin, intralipid or bilirubin up to concentration based on CLSI guideline EP07, severel drug intereferences tested negative QUANTA Flash circulating Calprotectin: microbial contamination, heat treatment, bilirubin ≥ 1 mg/ml, human IgG ≥ 70 mg/ml, Rheumatoid factor ≥ 196.5 IU/ml, cholesterol ≥ 332.5 mg/dl, triglyceride ≥ 1000 mg/dl |
|  | Sample processing (time until centrifugation): should not exceed 2 hours |
|  | Sample storage (stability) |
|  | Repeated freeze-thaw cycles (stability) |
|  | Wrong parameter requested, e.g. S100 protein (S100A or S100B as tumor markers) instead of S100A8/A9 |
| Analytical phase | Assay specific problems: wrong storage, expiration date |
|  | Non-compliance to manufacturer's protocol |
|  | False-low results, e.g. due to hook effect: avoid by use of additional dilution(s) |
| Interpretation | Comparison of different laboratory units (e.g. ng/ml, µg/ml) |
|  | Results obtained by different assays |
|  | Results out of linear range: Define linear range in standard curve |
|  | Prefer a laboratory that participates in ringtests for quality management |

**Supplemental table S3:**

Recommendation for troubleshouting and refinement for measurement of serum calprotectin in human samples according to used assay.

**Supplemental figure S1: Bland–Altman analysis of agreement between serum calprotectin assays.**

Bland–Altman plots show the agreement between calprotectin concentrations obtained with the extended-dilution Ref. BHL EK and (A) BHL EK, (B) R&D, (C) BioLegend, (D) BMA, (E) sCAL, (F) GCAL, and (G) Werfen. Calprotectin concentrations were log10-transformed before analysis. For each sample, the difference between the log10-transformed values of the BHL EK comparator and the respective comparison assay (A – G) is plotted against their mean. (H), (I) and (J) show comparisons of the automated assays. The solid horizontal line indicates the mean difference (bias), and the dotted lines indicate the 95% limits of agreement (bias ± 1.96 SD).

G

F

E

D

C

B

A

I

H

J

**Supplemental figure S2:**

Correlation of current Reference BHL EK (x-axis) with historical BHL EK (y-axisI). r = 0.99 (p < 0.0001).
